# Efficacy of proprioceptive training on the recovery of total joint arthroplasty patients: a meta-analysis

**DOI:** 10.1186/s13018-020-01970-6

**Published:** 2020-11-03

**Authors:** Wen-chao Zhang, Deng Xiao

**Affiliations:** Department of Neurorehabilitation, The Affiliated Rehabilitation Hospital of Chongqing Medical University, No. 50, Wenhuaqicun, Chongqing, Jiulongpo district China

**Keywords:** Osteoarthritis, Proprioceptive training, Meta-analysis

## Abstract

**Background:**

Optimal balance control is of paramount importance for function recovery after total joint arthroplasty (TJA). The study objective of this meta-analysis was to assess the short- and mid-term effects of proprioceptive and balance training for patients undergoing TJA.

**Methods:**

Electronic searches were conducted from PubMed, Cochrane library, and Embase databases to identify eligible RCTs through May 2020. Standard mean difference (SMD) with 95% confidence interval (95%CI) was applied to calculate pooled effect estimates between proprioceptive and balance training and control group. Main outcomes were self-reported functionality, balance, pain, quality of life, and function (range of motion).

**Results:**

Seven randomized controlled trials were finally included in this meta-analysis. Pooled results found that balance and proprioceptive trainings have a positive role in improving self-reported functionality at short-term after TJA. Moreover, balance and proprioceptive trainings were associated with an increase of the balance at short- and mid-term after TJA. These results were further confirmed by subgroup analysis between preoperative and postoperative administration of balance and proprioceptive trainings.

**Conclusion:**

Our meta-analysis suggests that balance and proprioceptive trainings after TJA improved self-reported functionality and balance. These improvements were maintained at mid-terms. More research is needed to confirm balance and proprioceptive trainings for pain and quality of life for TJA.

## Background

Osteoarthritis (OA) is the most prevalent joint disease characterized by joint pain, tenderness, and stiffness that finally can lead to the loss of joint function [1–3]. Joint arthroplasty is offered when conservative therapy does not alleviate severe pain or dysfunction of the joint [4]. Hip and knee arthroplasty costs exceeded US$1087.43 million [5]. These numbers are expected to rise further due to increasing proportion of aging population and obese population [6].

The results of joint arthroplasty procedures are overall satisfactory [7]. However, there are some barriers for balance and postoperative function [8, 9]. One study that investigated postoperative function in patients after total knee arthroplasty (TKA) indicated that postoperative pain and lack of effectiveness exercise are key causes of prolonged recovery following joint arthroplasty [10]. Among these adversities are exposure to deficits in the proprioceptive system, and thus, it is difficult to main postural control [11].

To optimize joint arthroplasty, it is necessary to explore additional effective rehabilitation protocols. Proprioceptive training has been investigated as a mean to achieve complete rehabilitation. Studies examining proprioceptive training following joint arthroplasty have produced conflicting results. Jogi et al. [12] revealed that proprioceptive training protocol resulted in significantly greater improvements in function than that of typical exercises alone. However, disagreements still remain about that proprioceptive training did not have any benefits for joint arthroplasty patients [13].

Therefore, this review and meta-analysis systematically assessed the effect of proprioceptive training in patients undergoing total joint arthroplasty (TJA), in terms of post-operative self-reported functionality, balance, pain, quality of life, and postoperative function (range of motion).

## Methods

This meta-analysis was carried out in accordance with the recommendations of the Cochrane Collaboration and the Preferred Reporting Items for Systematic Reviews and Meta-Analyses (PRISMA) guidelines [14].

### Search strategy

Three electronic databases (PubMed, Cochrane library, and Embase databases) were used for article retrieval. Two reviewers independently searched these databases from inception to May 20, 2020. The search criteria “proprioceptive training”, “balance exercise”, “education”, “training”, “total knee arthroplasty/replacement”, “TKA”, “TKR”, “total hip arthroplasty/replacement”, “THA”, and “THR” were used in keywords for search. No restrictions were applied for the country, year of publication, publication status, type of study, or language. And the reference lists were also manually reviewed to find relevant studies that were not found during the database searches.

### Inclusion criteria

Inclusion criteria were based on the PICOS strategy: (P) adult patients with knee or hip joint degenerative disease and received TKA or THA; (I) intervention group was proprioceptive training; (C) comparison group was standard procedure or no intervention; (O) outcomes including self-reported functionality (SRF), quality of life (QoL), Biodex Balance System (BBS), pain (P), balance (B), and knee function (KF); (S) randomized controlled trials (RCTs).

Exclusion criteria for this meta-analysis were as follows: (1) studies without available data; (2) abstract of full text was not available; (3) non-RCTs; (4) review manuscripts; (5) non-relevant studies.

### Data extraction

General information of the included studies was extracted by using a standardized data extraction form and recorded into Excel (Microsoft Excel 2019, Microsoft, Redmond, WA, USA). Study data extracted included first author, publication year, participants (total knee arthroplasty, total hip arthroplasty, or both), number of patients, mean age of patients, female patients (%), study type, intervention, comparison, and outcomes. If differences in opinion existed, the diagnosis was decided by their discussion and to reach total agreement.

### Risk of bias

Two reviewers (Wen-chao Zhang and Deng Xiao) independently complete the process of quality assessment. The following aspects will be assessed: random sequence generation (selection bias), allocation concealment (selection bias), blinding of participants and personnel (performance bias), incomplete outcome data (attrition bias), selective reporting (reporting bias), and other bias. Each aspect was classified as “low,” “high,” or “unclear” according to Cochrane Collaboration Handbook recommendations.

Finally, quality of evidence was generated according to GRADE (Grading of Recommendations Assessment, Development and Evaluation) methodology and performed by utilizing the GRADE Pro GDT software. A total of four categories were used: high, moderate, low, or very low.

### Statistical analysis

The statistical analysis was performed using the Stata 12.0 software (Stata Corp LLC, College Station, TX, USA). Pooled data were assessed by standard mean difference (SMD) with 95% confidence intervals (CIs), and *P* < 0.05 was considered statistically significant. The *I*^2^ value was used to assess the degree of heterogeneity (*I*^2^ of 0% indicated no heterogeneity; *I*^2^ > 50%, low heterogeneity, and *I*^2^ ≥ 50%, high heterogeneity). Publication bias was assessed by visual inspection of the funnel plot, and we accepted that a symmetrical funnel plot was likely to indicate low publication bias. A subgroup analysis was performed by duration of follow-up: early period (6–12 weeks), mid-term (6–12 months), and long-term (> 12 months). Another subgroup analysis was conducted by the timing of the intervention: preoperative intervention and postoperative intervention.

## Results

### Search results

**Fig. 1:**
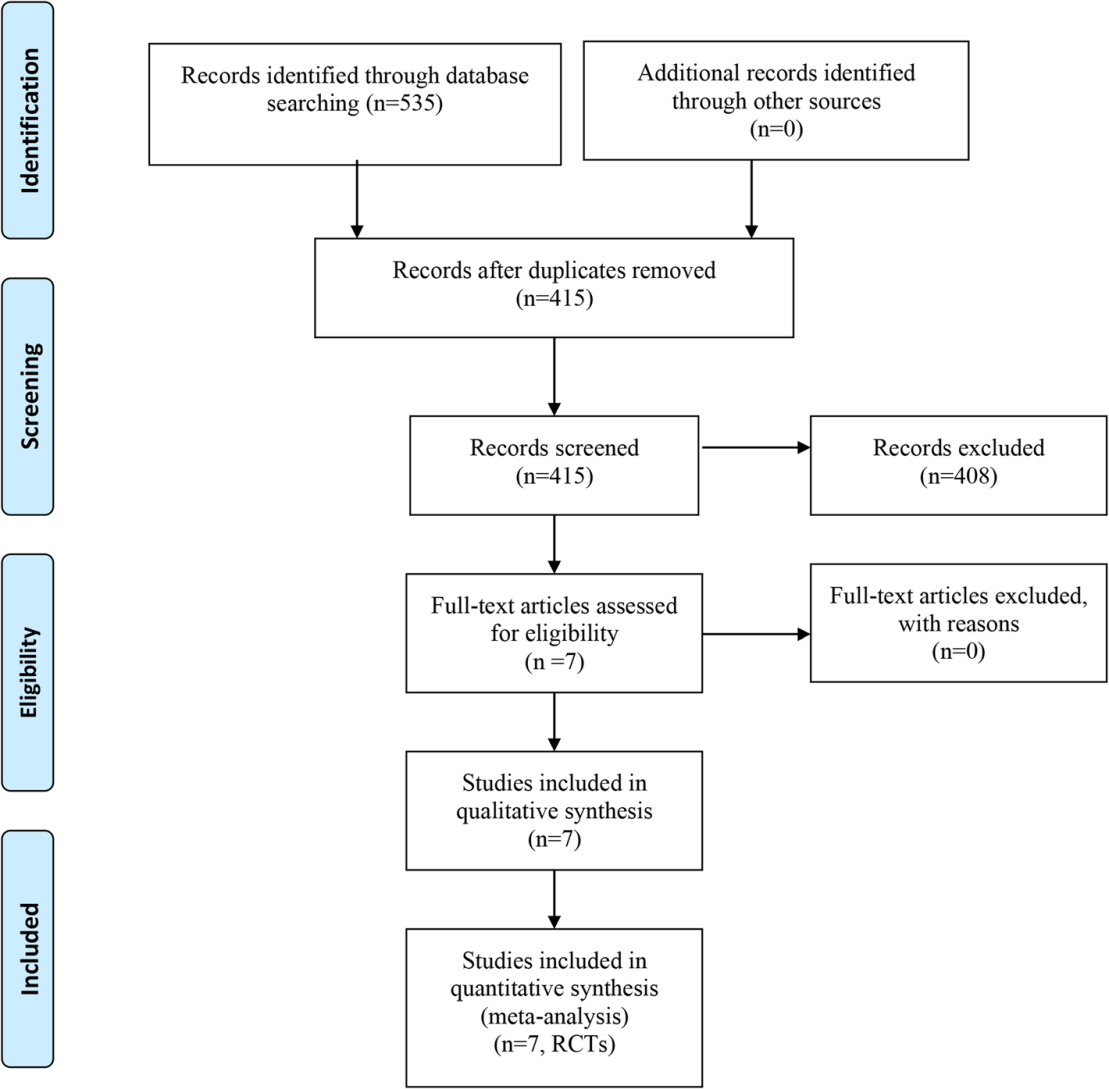
The PRISMA flowchart regarding the study selection process

The initial literature search retrieved 535 relevant articles. After duplicates were discarded, 415 studies were screened. After reviewing the abstracts, 408 articles were excluded because they did not meet inclusion and exclusion criteria; therefore, leaving 7 studies that matched the selection criteria and were suitable for meta-analysis (Fig. 1) [12, 15–21]. A total of 567 (balance and proprioceptive trainings, 282; control group, 285) patients were enrolled in the studies; the general characteristic of the included patients is summarized in Table 1. These reports were published between 2010 and 2018 for evaluation. The mean size of patient sample was 70 (range from 35 to 165). One study included only total hip arthroplasty patients, 4 included only total knee arthroplasty patients, and 2 included both total hip arthroplasty and total knee arthroplasty patients. Control group interventions mainly consisted of strengthening and range of motion exercise, standard program, knee school, usual RHB, and educational package. The interventions were the same as those in the corresponding control groups, but additionally included an experimental balance training, i.e., warm-up, stretching and balance exercises, NEMEX program + knee school, strengthening and range of motion exercise + 3 balance exercises, sensorimotor training, and standard RHB + balance exercise.

**Table 1:** General characteristic of the included studies

Author	Participants	No. of patients	Mean age (year)	Female patients (%)	Study	Intervention	Comparison	Outcomes
Jogi et al. [12]	TKA and THA	54	66.4	33.0	RCT	Strengthening and ROM exercise + 3 balance exercises	Strengthening and ROM exercise	SRF: WOMAC B: ASBC BBS, TUG
Bitterli et al. [15]	THA	80	66.8	38.8	RCT	Sensorimotor training	No intervention	SRF: SF-36, WOMAC Qol: SF-36 B: Biodex Balance System
Gstoettner et al. [16]	TKA	38	69.6	78.9	RCT	Warm-up, stretching and balance exercises	No intervention	SRF: WOMAC, KSS B: Biodex Balance System P: WOMAC O: walking 60 m, stairs up
Huber et al. [17]	TKA	45	70.3	46.6	RCT	NEMEX program + knee school	Knee school	SRF: KOSS B: TUG, Chair Stand Test KF:ROM P: VAS
Liao et al. [18]	TKA	113	72.1	35.4	RCT	Standard RHB + balance training	Usual RHB	SRF: WOMAC B: SLSB, FRT, TUG O:10MWT,30s timed chair stand test, climb stairs
Piva et al. [19]	TKA	35	68.4	71.4	RCT	Standard RHB + balance exercise	Standard program	SRF: WOMAC, LEFS B: SLSB P: VAS, WOMAC O: Gait speed, get up test
Roig-Casasús et al. [20]	TKA	37	73.4	67.5	RCT	Standard RHB + 20’ of dynamometric platform exercise	Usual RHB	B: BBS, TUG, FRT, Platform measures
Villadsen et al. [21]	TKA and THA	165	67.4	55.7	RCT	NEMEX program + educational package	Educational package	SRF: KOOS, HOOS B: 5 times “get up” P: VAS Qol: EQOL-5D O: 20MWT, KF in 30’

*TKA* total knee arthroplasty, *THA* total hip arthroplasty, *RCT* randomized controlled trials, *SRF* self-reported functionality, *B* balance, *P* pain, *KF* knee function, *O* other outcomes, *RHB* rehabilitation, *SLSB* single leg standing balance, *FRT* Functional Reach Test, *TUG* Time Up and Go, *WOMAC* Western Ontario and McMaster Universities Osteoarthritis, *LEFS* Lower Extremity functional Scale, *VAS* visual analog scale, *ROM* range of movement, *BBS* Berg Balance Scale, *ABC* activities specific balance scale, *KSS* Knee Society Score, *KOOS* Knee Injury and Osteoarthritis Score, *EQOL* Euro Quality of Life 5 Dimensions, *MWT* minutes-meters walking test

### Risk of bias

**Fig. 2:**
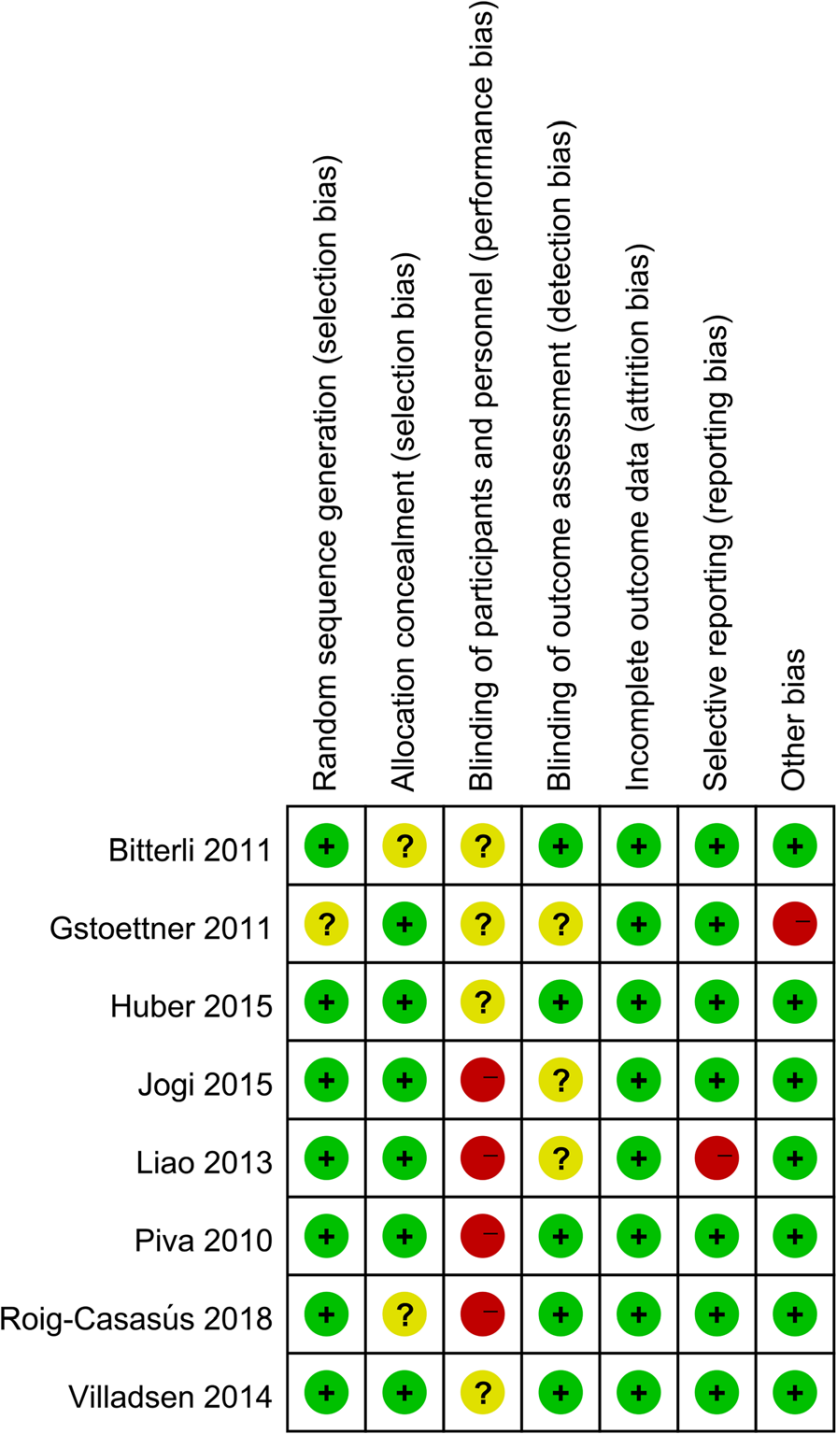
Risk of bias summary for the included studies. Plus sign indicates low risk of bias; minus sign indicates high risk of bias; question mark indicates unclear risk of bias

**Fig. 3:**
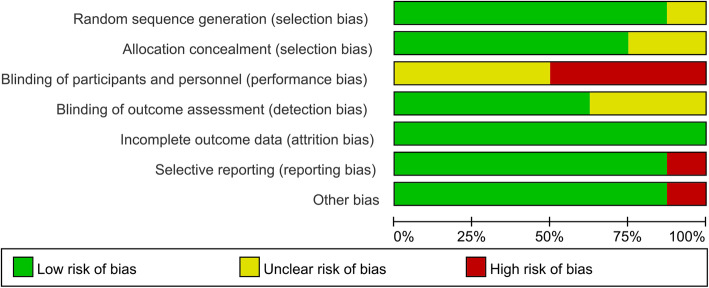
Risk of bias graph for the included studies

The risk of bias summary and risk of bias graph for included 7 RCTs are shown in Figs. 2 and 3. In general, 5 studies (Jogi et al. [12], Gstoettner et al. [16], Liao et al. [18], Piva et al. [19], and Roig-Casasús et al. [20]) were considered to have high risk of bias while the other 3 have unclear risk of bias. Seven trials had a low risk of bias in random sequence generation, and six trials had a low risk of bias in allocation concealment. Four studies had a high risk of bias in blinding of participants and personnel. One study had a high risk of bias in selective reporting, and one study had a high risk of bias in other bias.

### Results of meta-analysis

#### Balance and proprioceptive trainings for functional outcomes at early postoperative

**Fig. 4:**
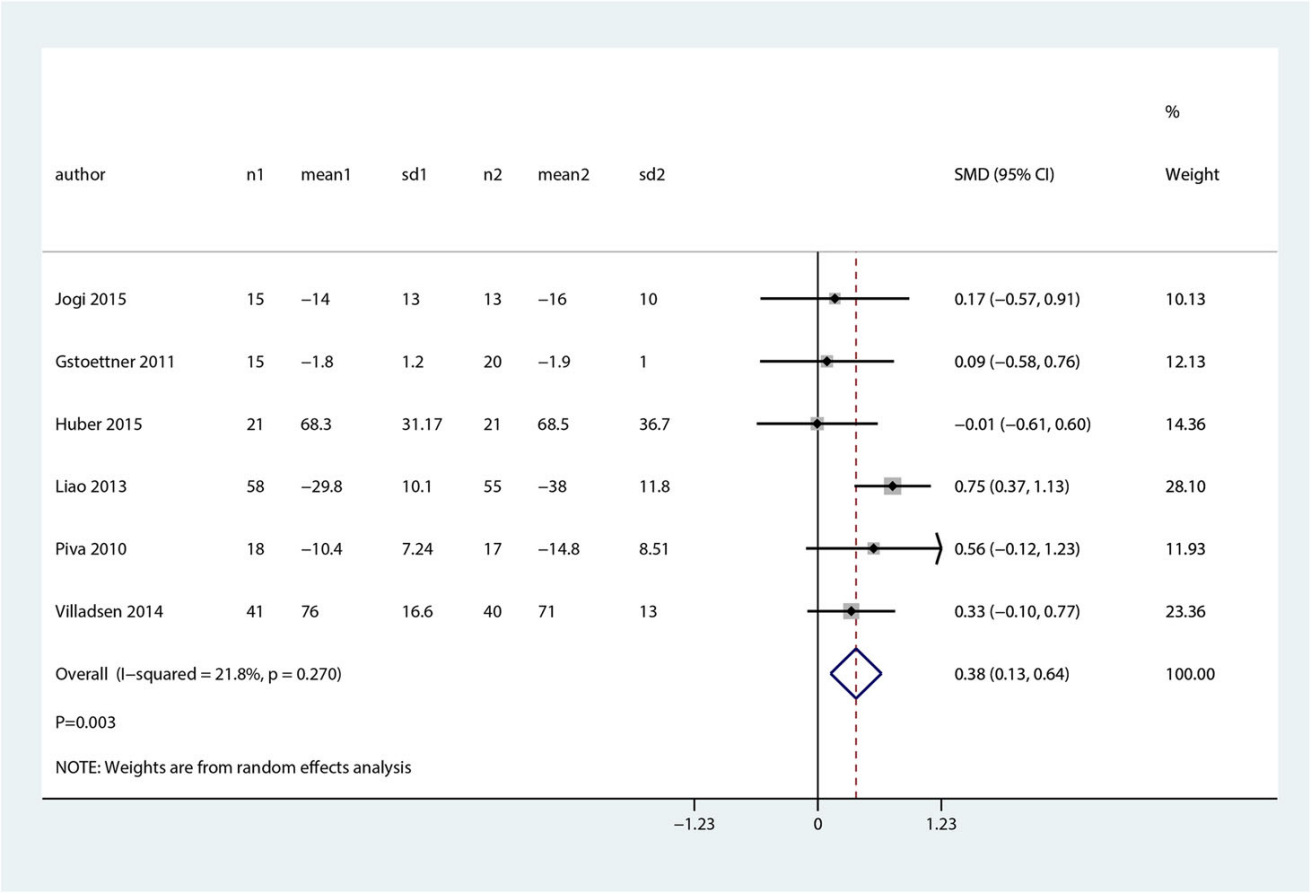
Balance and proprioceptive trainings and control on self-reported functionality

The breakdown number of studies for the effects between balance and proprioceptive trainings and control groups in patients after TJA on self-reported functionality at early postoperative (SMD 0.38; 95% CI 0.13 to 0.64; *P* = 0.003, Fig. 4).

**Fig. 5:**
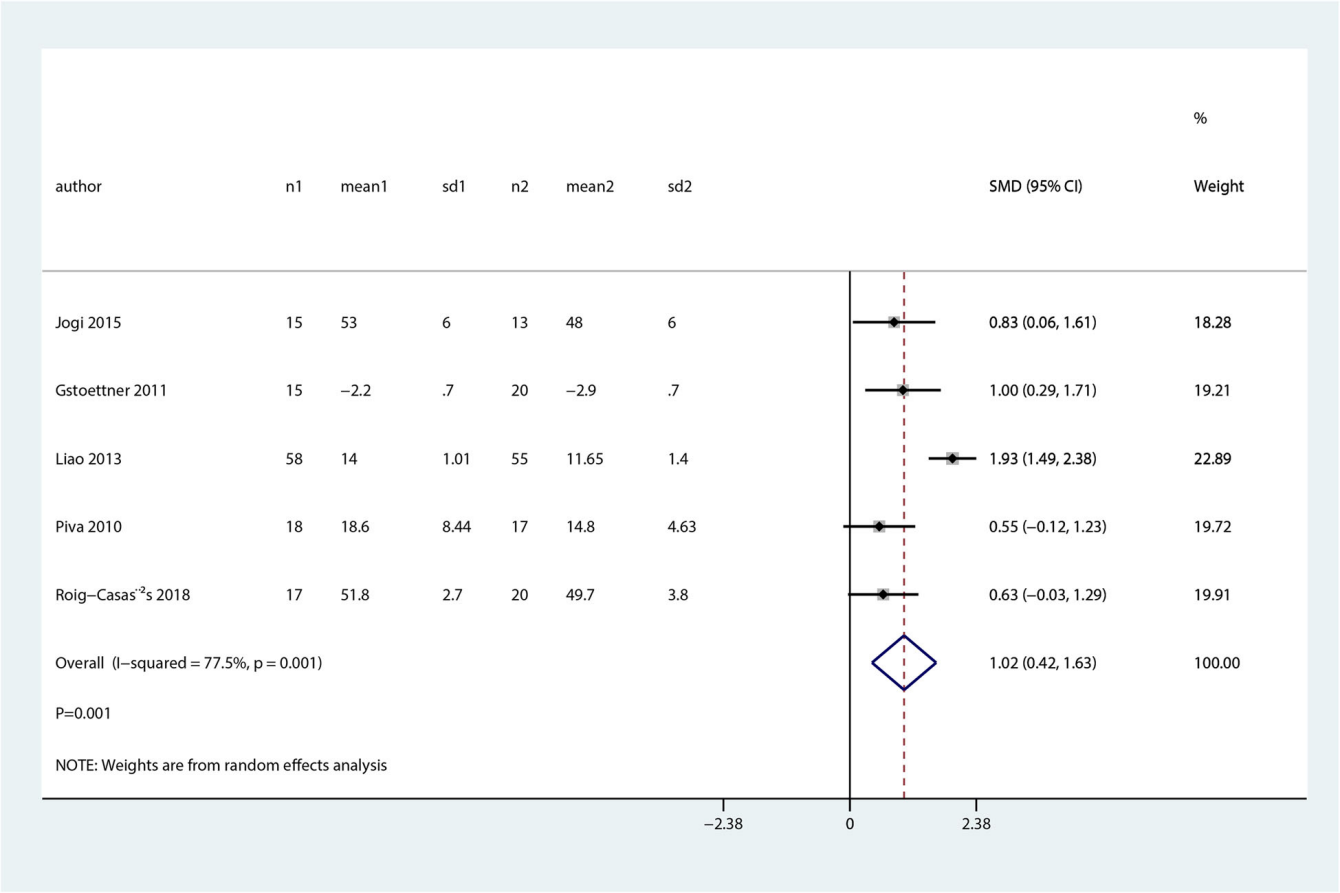
Forest plot of proprioceptive trainings and control in terms of balance

**Fig. 6:**
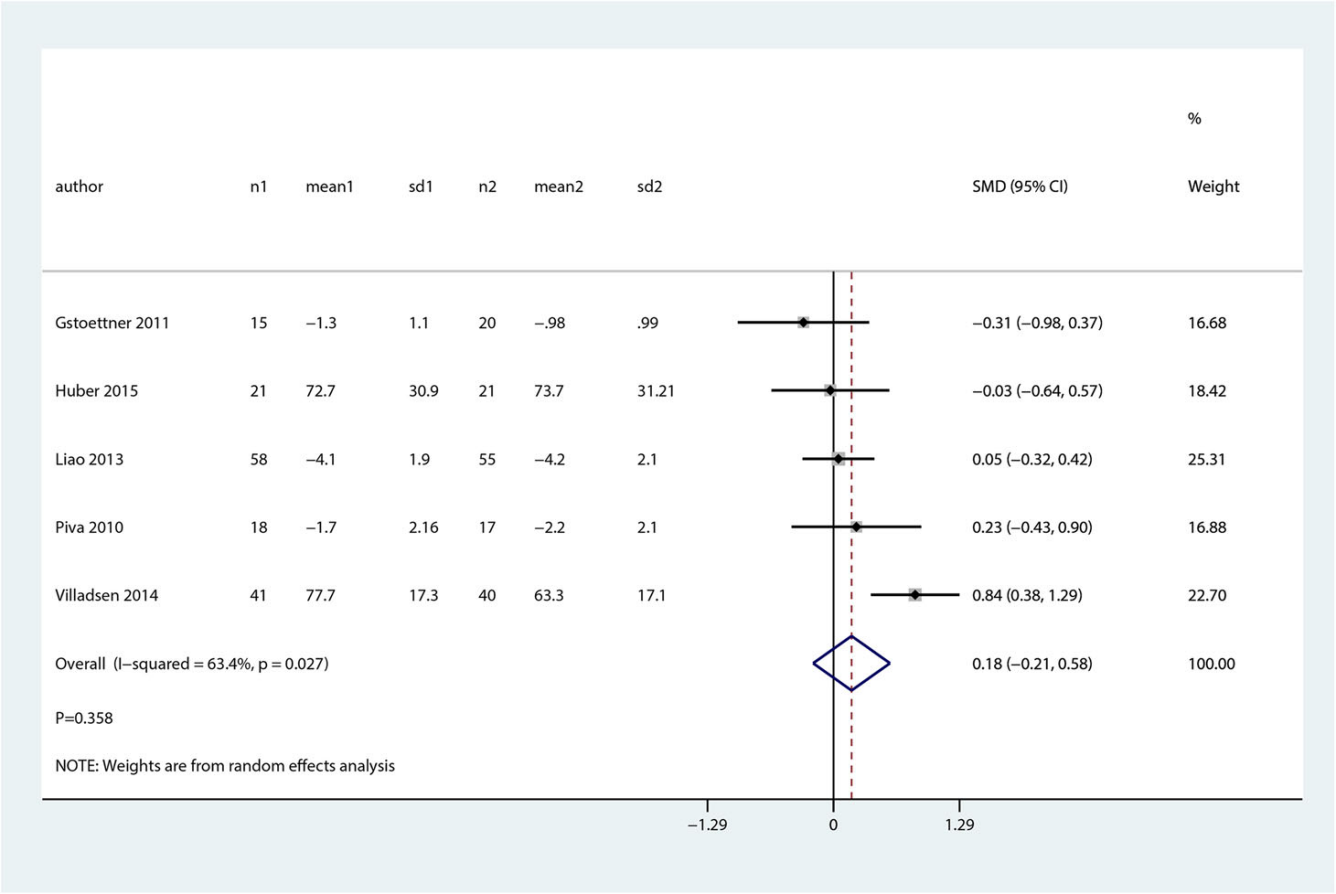
Forest plot of proprioceptive trainings and control in terms of pain scores

We noted that balance and proprioceptive trainings were associated with higher balance at early postoperative (SMD 1.02; 95% CI 0.42 to 1.63; *P* = 0.001, Fig. 5) as compared with control group, while no significant differences between groups for pain scores (SMD 0.18; 95% CI − 0.21 to 0.58; *P* = 0.358, Fig. 6).

**Fig. 7:**
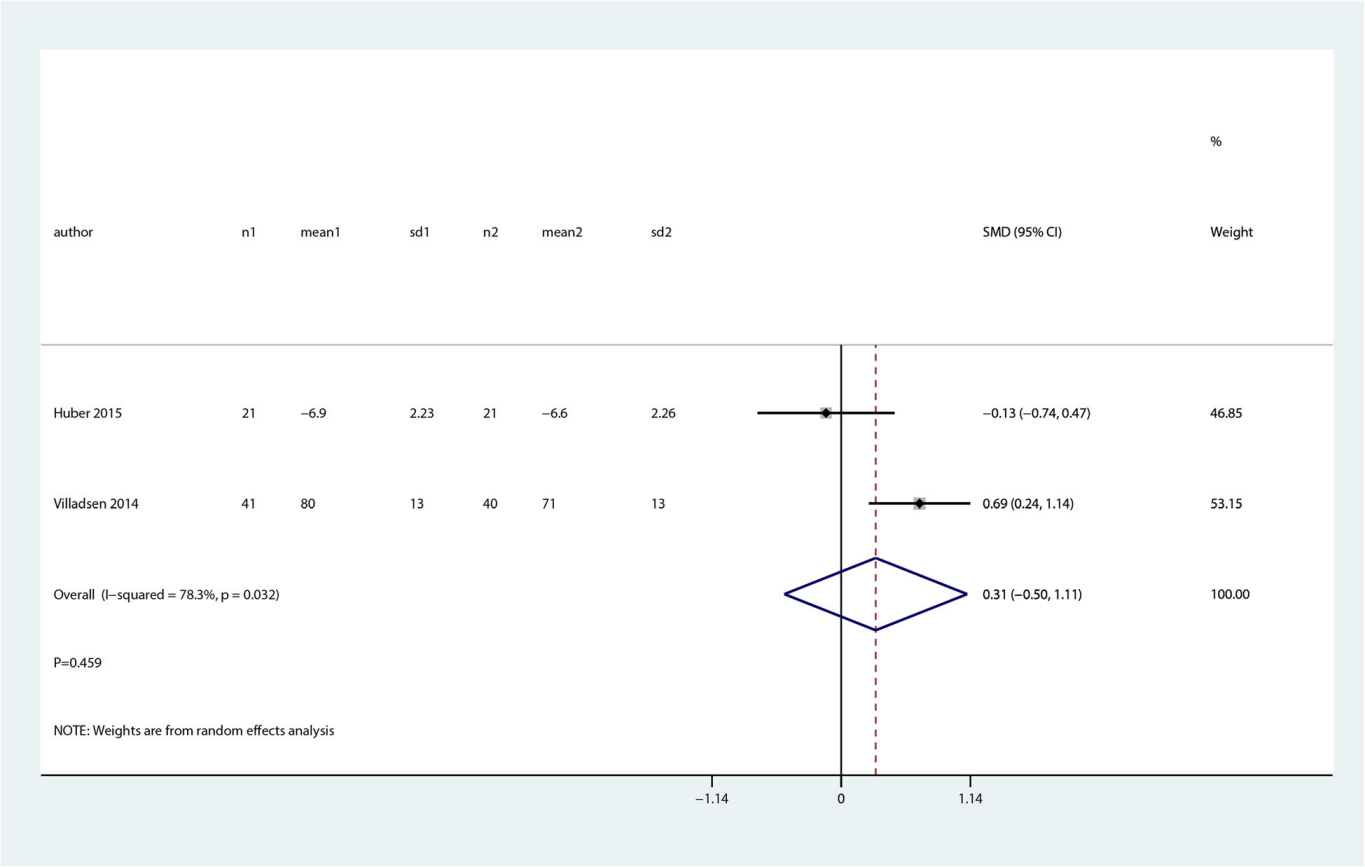
Forest plot of proprioceptive trainings and control in terms of quality of life

**Fig. 8:**
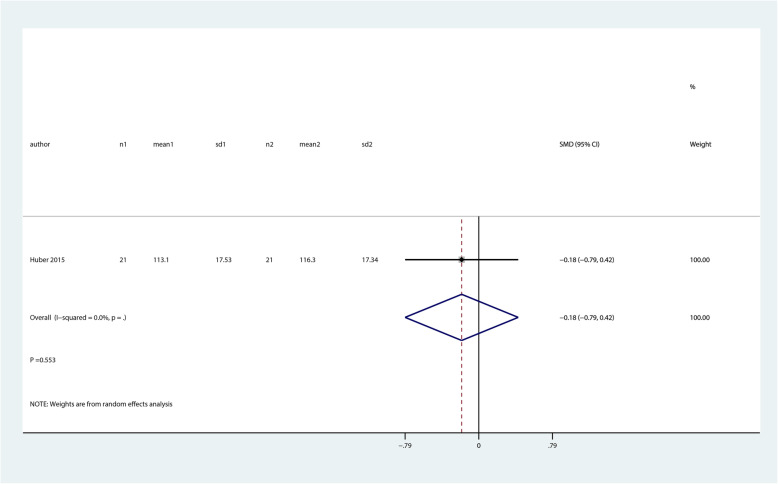
Forest plot of proprioceptive trainings and control in terms of function (ROM)

There were no significant differences between balance and proprioceptive trainings and control groups for quality of life (SMD 0.31; 95% CI − 0.50 to 1.11; *P* = 0.459, Fig. 7) and function (ROM, SMD − 0.18; 95% CI − 0.79 to 0.42; *P* = 0.553, Fig. 8).

#### Balance and proprioceptive trainings for functional outcomes at mid-term

**Fig. 9:**
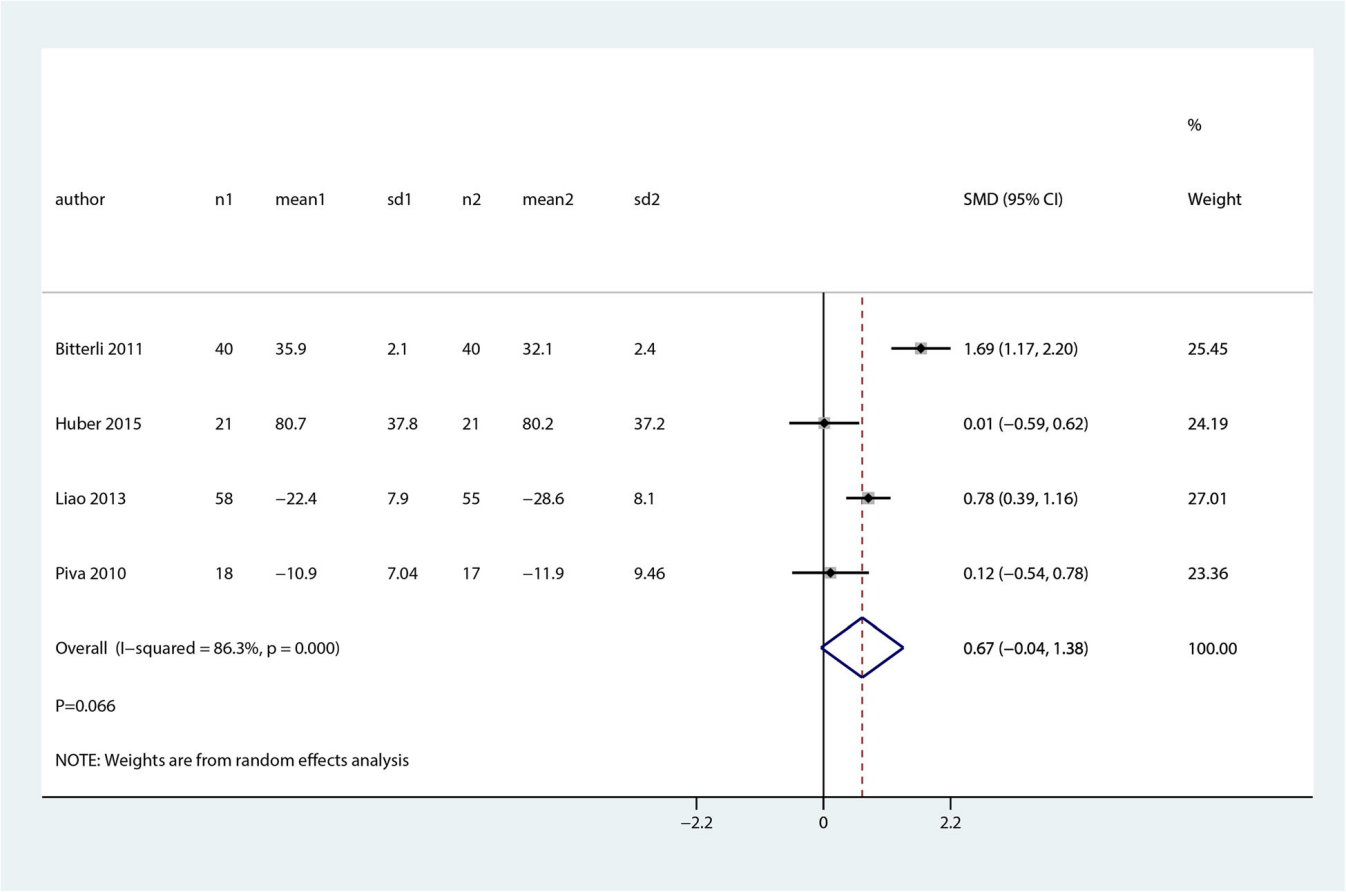
Forest plot of proprioceptive trainings and control in terms of self-reported functionality at mid-term

Data for the effect of balance and proprioceptive trainings versus control groups on the self-reported functionality at mid-term were available in 4 studies. There was no significant difference between balance and proprioceptive trainings and control groups in terms of the self-reported functionality at mid-term (SMD 0.67; 95% CI − 0.04 to 1.38; *P* = 0.066, Fig. 9).

**Fig. 10:**
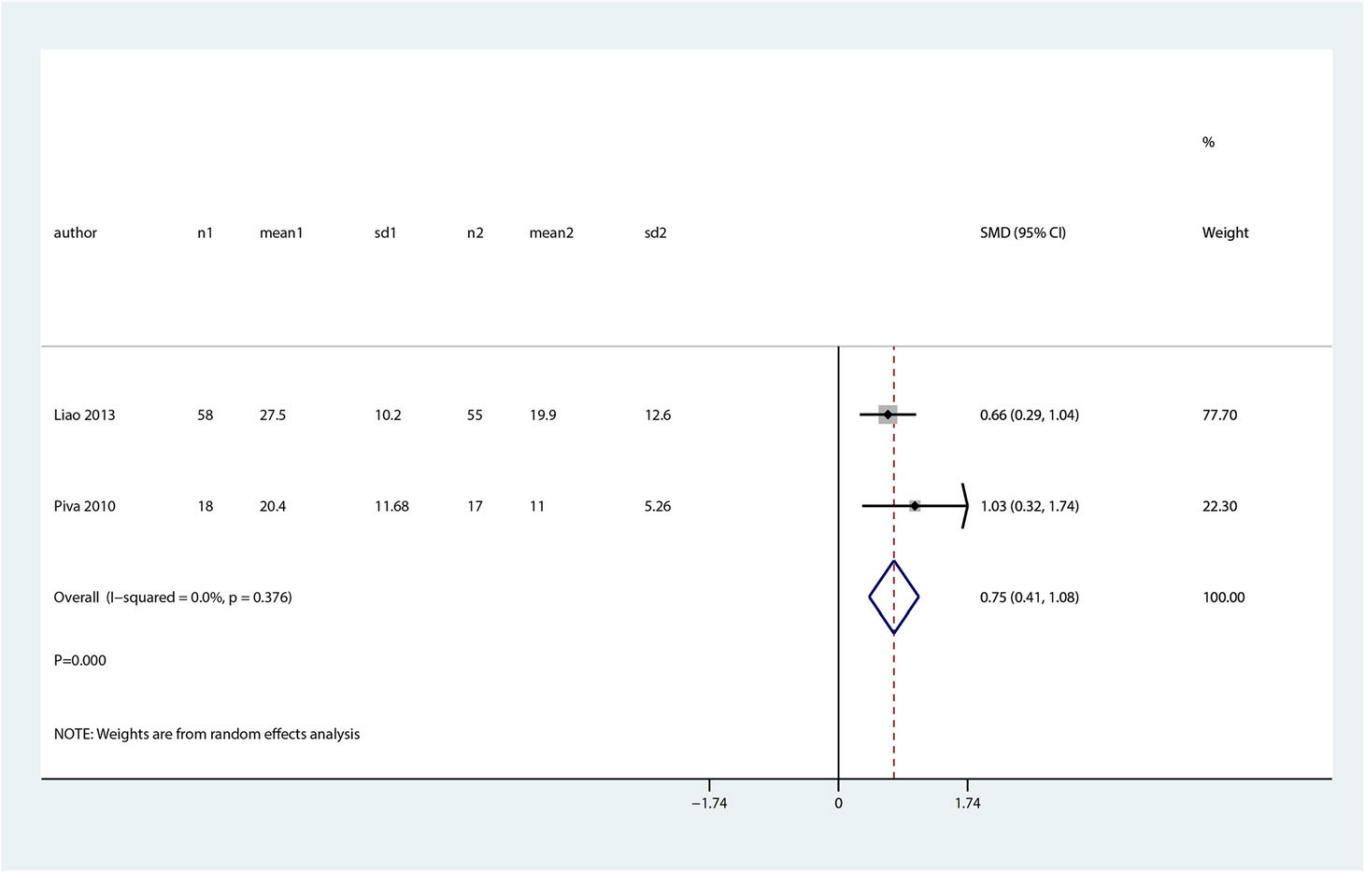
Forest plot of proprioceptive trainings and control in terms of balance at mid-term

**Fig. 11:**
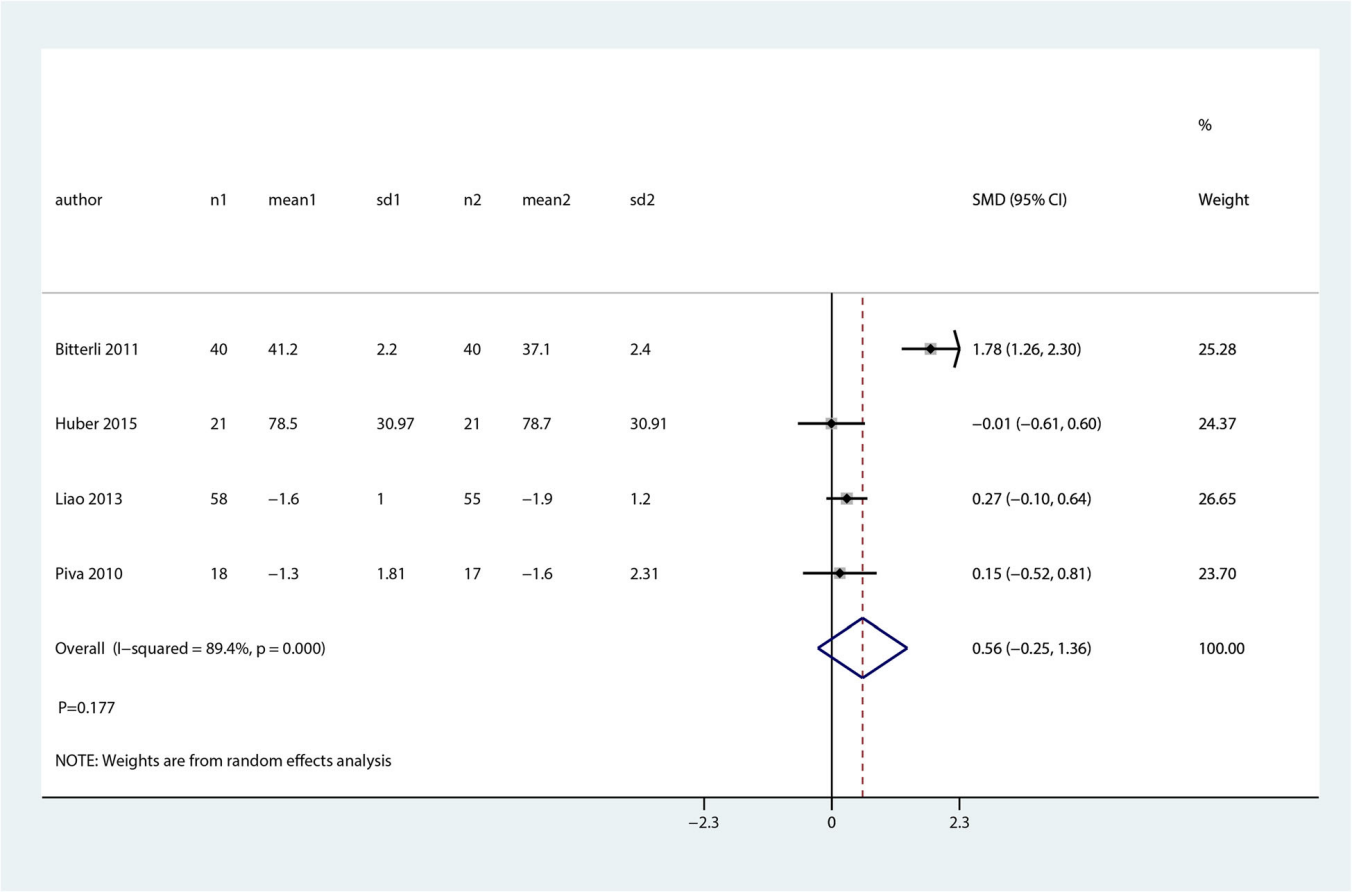
Forest plot of proprioceptive trainings and control in terms of pain at mid-term

**Fig. 12:**
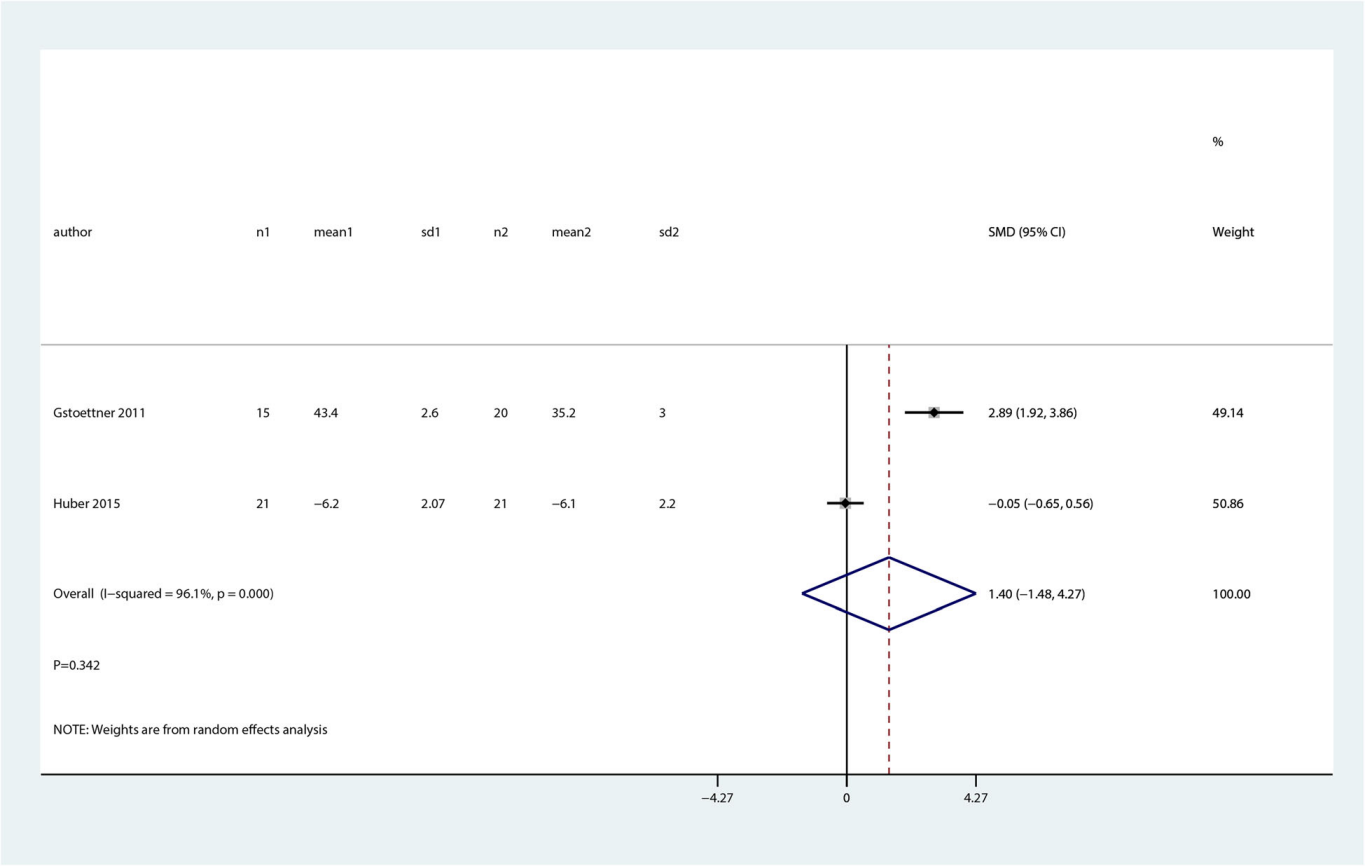
Forest plot of proprioceptive trainings and control in terms of quality of life at mid-term

We noted that balance and proprioceptive trainings are associated with higher balance at mid-term as compared with control group (SMD 0.75; 95% CI 0.41 to 1.08; *P* = 0.000, Fig. 10). There was no significant difference between balance and proprioceptive trainings and control groups in terms of the pain (SMD 0.56; 95% CI − 0.25 to 1.36; *P* = 0.177, Fig. 11) and quality of life (SMD 1.40; 95% CI − 1.48 to 4.27; *P* = 0.342, Fig. 12) at mid-term.

#### Subgroup analysis

Results of the subgroup analysis are presented in Table 2. The findings of pain, ROM, QoL, and balance were consistent in all subgroup analyses except for the self-reported functionality. In an analysis stratified by timing of training (preoperative vs postoperative), the summary SMD from postoperative interventions (SMD 0.61; 95 % CI 0.31–0.92, *P* = 0.000) showed that there was statistically significance between balance and proprioceptive trainings and controls, but no significant difference in preoperative intervention (OR 0.19; 95% CI − 0.12–0.50, *P* = 0.236).

**Table 2:** Subgroup analysis for the outcomes

Subgroup	Early post-operative effects				Mid-term effects			
	Trials	Sample	SMD (95% CI)	*P* value	Trials	Sample	SMD (95% CI)	*P* value
Preoperative interventions								
Pain	3	158	0.20 (− 0.53, 0.92)	0.593	2	122	0.89 (− 0.86, 2.65)	0.317
ROM	1	42	− 0.18 (− 0.79, 0.42)	0.553				
QoL	2	123	0.31 (− 0.50, 1.11)	0.459	2	77	1.40 (− 1.48, 4.27)	0.342
Self-reported functionality	3	158	0.19 (− 0.12,0.50)	0.236	2	122	0.86 (− 0.78, 2.50)	0.305
Balance	1	35	1.00 (0.29, 1.71)	0.006				
Postoperative interventions								
Pain	2	148	0.09 (− 0.23, 0.41)	0.575	2	148	0.24 (− 0.08, 0.57)	0.143
ROM								
QoL								
Self-reported functionality	3	176	0.61 (0.31, 0.92)	0.000	2	148	0.51 (− 0.13, 1.14)	0.116
Balance	4	213	1.02 (0.26, 1.77)	0.008	2	148	0.75 (0.41, 1.08)	0.000
Overall effects								
Pain	5	306	0.18 (− 0.21, 0.58)	0.358	4	270	0.56 (− 0.25, 1.36)	0.177
ROM	1	42	− 0.18 (− 0.79, 0.42)	0.553				
QoL	2	123	0.31 (− 0.50, 1.11)	0.459	2	78	1.40 (− 1.48, 4.27)	0.342
Self-reported functionality	6	334	0.38 (0.13, 0.64)	0.003	4	270	0.67 (− 0.04, 1.38)	0.066
Balance	5	248	1.02 (0.42, 1.63)	0.001	2	148	0.75 (0.41, 1.08)	0.000

#### Grade profile evidence and publication bias

**Fig. 13:**
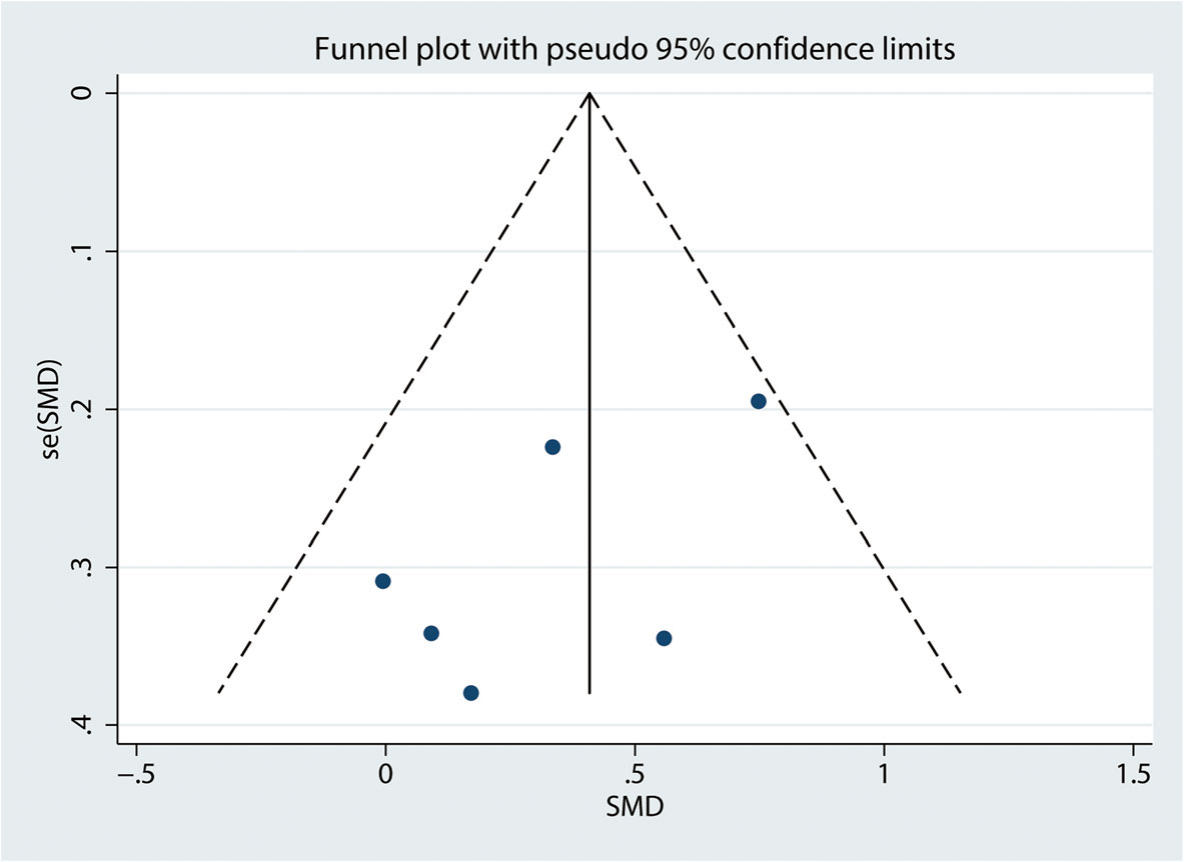
Funnel plot of self-reported functionality

The GRADE working group grade level of evidence is low for ROM and QoL and moderate for pain, self-reported functionality, and balance (Table 3). Publication bias was assessed by visual inspection of the funnel plots, and no distinct asymmetry was found (Fig. 13).

**Table 3:** Grade evidence of the outcomes

Outcomes	Relative effect (95% CI)	No. of participants (studies)	Certainty of the evidence (GRADE)
Pain	0.18 (− 0.21, 0.58)	306 (5)	Moderate
ROM	− 0.18 (− 0.79, 0.42)	42 (1)	Low
QoL	0.31 (− 0.50, 1.11)	123 (2)	Low
Self-reported functionality	0.38 (0.13, 0.64)	334 (6)	Moderate
Balance	1.02 (0.42, 1.63)	248 (5)	Moderate

## Discussion

This meta-analysis appraised the effects of balance and proprioceptive trainings in patients undergoing TJA. Based on the pooled effects, balance and proprioceptive trainings enhanced the early postoperative functional outcomes after TJA. What’s more, follow-up evaluations confirmed that the promotion effects of balance and proprioceptive trainings for balance were maintained at mid-term. In subgroup analysis, postoperative balance and proprioceptive trainings were associated with better functional outcomes in TJA patients.

A major strength of this meta-analysis is that we assessed the most important clinical outcome, self-reported functionality, and balance at early period and mid-term follow-up. Another strength of this meta-analysis was the good stability of the results, which is reflected in subgroup analysis.

Previous study has reported that proprioceptive inaccuracy is the main cause of functional deterioration. According to the theory mentioned above, well-targeted post-operative proprioceptive intervention can further enhance the patients’ functional performance and quality of life. An observational study has pointed out that game-based balance exercises could improve motor performance and postural control in long-term follow-up [22]. Kakavas et al. [23] reported that training can improve function and finally to optimize return to play in anterior cruciate ligament. These results suggest that proprioceptive intervention can also be administrated in TJA patients to improve postoperative function.

Another important finding from our analysis is that balance and proprioceptive trainings showed no benefit for pain, range of motion, and quality of life at early period and mid-term follow-up. Appropriately 30% of patients experience moderate to severe pain at 1 year after surgery [24]. Several mechanisms may underlie postoperative pain, including peripheral and central sensitization. Pain management is a critical but complex issue in the relief of acute pain, particularly important for functionally recovery for TJA patients. Kosek et al. [25] reported that exercise has no effects on the pain severity in osteoarthritis patients. These results are similar to our results and agree that the balance training has no effects on pain control in TJA patients.

Consistent with pain outcome, balance and proprioceptive trainings also have no effects on the quality of life after TJA compared with standard procedures. TJA itself could significantly improve the quality of life in TJA patients. There was no further improvement in balance and proprioceptive trainings than that of standard procedures. Due to the low number of these studies, more studies are needed to confirm and elucidate this finding. These results were clinically important as balance and proprioceptive trainings only have benefit for improving balance and self-reported functionality. These improvement effects were maintained at mid-term follow-up. Moreover, postoperative balance and proprioceptive trainings was superior than preoperative balance and proprioceptive trainings in terms of the balance and self-reported functionality. One important consideration was that TJA surgeries have vital influence on the proprioception. Thus, postoperative balance and proprioceptive trainings could improve the balance and functionality at some extent. Lee et al. [26] included a total of 8 RCTs and assessed the balance training after hip fracture. Results suggested that balance training significantly improve overall physical functioning and balance. And author also recommended that balance training should be specifically included in postoperative rehabilitation programs.

To prevent selection bias within this meta-analysis, only RCTs were included for final analysis. However, some potential limitations in this study were inevitable. Firstly, the duration, timing, and type of the balance and proprioceptive trainings differed across studies. This may affect the final results; in addition, some important data (long-term functional outcomes) were lacking, hampering analysis. Secondly, small sample size of the included studies may render the results underpowered. Thirdly, the heterogeneous outcomes were also a limitation. Subgroup analysis was performed to assess the robustness of results; however, not all planned subgroup analyses could be performed, due to insufficient data reporting. Thus, the heterogeneity could not always be explained. Fourth, the data for pain and other adverse effects was limited, and these results need for more studies to validate. Lastly, the clinical relevance of self-reported functionality and balance remain challenging due the short-term follow-up duration.

## Conclusion

Our meta-analysis suggests that balance and proprioceptive trainings after TJA improved self-reported functionality and balance. These improvements were maintained at mid-terms and postoperative balance and proprioceptive trainings. However, balance and proprioceptive trainings have no effects on pain relieving, functional recovery, and quality of life after TJA. Considering these effects of balance and proprioceptive trainings, more studies are needed to identify the balance and proprioceptive trainings for pain control and functional outcomes.

## Data Availability

We declare that the materials described in the manuscript will be free access to all of the reader in the included studies.

## Abbreviations

SMD: Standard mean difference
OA: Osteoarthritis
SRF: Self-reported functionality
QoL: Quality of life
BBS: Biodex Balance System
P: Pain
B: Balance
KF: Knee function
RCTs: Randomized controlled trials
GRADE: Grading of Recommendations Assessment, Development and Evaluation
CIs: Confidence intervals
